# Editorial: Valorization of food and agro-industrial waste: novel approaches and their applications

**DOI:** 10.3389/fnut.2023.1250816

**Published:** 2023-07-13

**Authors:** Kandi Sridhar, Minaxi Sharma, Baskaran Stephen Inbaraj

**Affiliations:** ^1^Department of Food Technology, Karpagam Academy of Higher Education, Coimbatore, India; ^2^CARAH ASBL, Ath, Belgium; ^3^Department of Food Science, Fu Jen Catholic University, New Taipei City, Taiwan

**Keywords:** valorization, food waste, agro-industrial waste, recovery, biorefinery, green methods, circular economy, by-products

Over the years, waste generated from agriculture and food industries has become a major issue in the entire agri-food production supply chain (1). By implementing the smart and sustainable valorization/revalorization strategies, food and agro-industrial waste can be reutilized for the production of low-cost value-added products for wider applications in pharmaceutical, cosmetic, food, and chemical industries (2). Thus, many pharmaceutical, cosmetic, food, and chemical industries adopted several food and agro-industrial waste management strategies, including reduce-reuse-recycle (3R), waste valorization/revalorization, and on/off-site food waste treatments. In this regard, to improve scientific knowledge and understanding of food and agro-industrial waste management strategies, a group of interdisciplinary experts (Dr. Baskaran Stephen Inbaraj, Dr. Kandi Sridhar, and Dr. Minaxi Sharma) in agri-food waste valorization, bioactive compounds, and value-added bio-based products organized a special Research Topic aiming at “*Valorization of food and agro-industrial waste: novel approaches and their applications*.” The objectives of this special Research Topic were to encourage the submission of high-quality scientific original research papers and state-of-the-art reviews that cover novel approaches to valorize food and agro-industrial waste/by-products and their potential application in the production of biorefineries, biochemicals, biodegradable materials, bioactive compounds, and food product development. We actively participated in organizing this special Research Topic by promoting and inviting global experts in the field, performed careful selection on submission, and expedited the single blind peer-review process. High-quality manuscripts were received for consideration and possible rigorous peer-review process. This Research Topic includes six high-quality publications dealing with different aspects of food/agro waste valorization and their potential applications, which can help to increase the scientific knowledge and advancement of this field.

A study by Rudrapal et al. investigated the biological activities of vasicine, a potent alkaloid found in *Adhatoda vasica*, which is a traditional medicinal herb used for various ailments. Vasicine was found to have antimicrobial, antioxidant, anti-inflammatory, antidiabetic, antiviral, and anticancer properties, as demonstrated by different biological and pharmacological assays, as well as *in silico* techniques. The compound showed good binding affinity with active binding sites of various enzymes and receptors involved in different human diseases. Likewise, another study by Venkata et al. used the bioactive constituents of *Andrographis paniculata* to minimize plant-based waste and investigated the potential use of andrographolide (AG) for the treatment of neurodegenerative illnesses. Using microarray expression profiling, eight differentially expressed genes (DEGs) associated with AG were identified. Andrographolide nanoparticles (AGNPs) were also prepared to enhance bioavailability and their neuroprotective effect against PTZ-induced kindling epilepsy was investigated using network pharmacology and docking studies. Results showed that AG and AGNPs significantly reduced kindling score and reversed oxidative damage in rats. Both the studies concluded that the bioactive compounds extracted from plant sources can be effective for treating a wide range of human diseases.

Giorni et al. studied the application of agri-food chain by-products in inhibition of mycotoxigenic species and related mycotoxins. The extracts derived from pear and grape marc were shown to exert significant inhibitory effect on the growth of *Aspergillus flavus* and *A. carbonarius* with reductions ranging from 45 to 47 % and 21 to 51 %, respectively. Additionally, extracts from grape stalk, pear, and grape marc showed a profound influence in inhibiting the growth of *Fusarium graminearum*. The extracts were also shown to exhibit inhibitory effects against various other mycotoxins. Likewise, another study by Maia et al. used post-harvest biomass residues from native European *Lupinus* species (straw and pod shells) and evaluated the production yield, nutritive value, and alkaloid content of straws and pod shells for potential application as animal feed. The results showed that lupin biomass residues had higher crude protein and lignin content compared to cereal straws commonly used in ruminant feeding. The study also suggested that consuming biomass residues with alkaloid content up to 85 % of dry matter intake is not expected to have negative effects on small and large ruminant species. Overall, both the study highlighted the potential of utilizing agro waste for the development of natural and environmentally friendly alternatives and promoting sustainability in a circular economy approach.

A review submitted by Zhang et al. provided novel approaches and perspectives for the efficient utilization of bioactive micro/macronutrients from food waste. This paper also reviewed the economic and technical guidance for the utilization of food waste. The study suggested that the use of hydrothermal treatment can be considered as a more economical and energy-integrated process to produce energy-dense fuels and valuable chemicals. This is essential information to be considered while selecting food-waste treatment methods to yield more value-added products and minimize the energy needed. Another review conducted by a group of researchers focused on the valorization of fruit waste for the development of fruit-based value-added functional products (Pathak et al.). In this context, the review aimed at sustainable utilization of jackfruit waste to produce value-added products. The authors concluded that the jackfruit waste is also an excellent source for a production of eco-friendly industrial products, such as biofuels, biomaterials, pharmaceuticals, and food and feed additives. Authors further suggested the use of more genetically and biotechnologically advanced strategies for the effective utilization of jackfruit waste.

Collectively, afore-mentioned studies clearly presented the different strategies for food/agro waste valorization and development of value-added products. We strongly believe that the presented articles create a source of information on food/agro-waste utilization approaches. Besides, these articles are open access publications (copyright © 2022 and/or copyright © 2023 by authors) distributed under the terms and conditions of the Creative Commons Attribution (CC BY) license to reach a wider audience and expand their knowledge. The vision behind this special Research Topic further opens up opportunities to explore the sustainable food/agro waste valorization and development of low-cost value-added products, which will be continued in future special Research Topics.

## Author contributions

KS: data curation, methodology, formal analysis, software, writing—original draft, validation, and visualization. MS and BS: conceptualization, data curation, writing—review and editing, supervision, visualization, and validation. All authors contributed to the article and approved the submitted version.
